# Behavioral and Brain Reactivity Associated With Drug-Related and Non-Drug-Related Emotional Stimuli in Methamphetamine Addicts

**DOI:** 10.3389/fnhum.2022.894911

**Published:** 2022-06-24

**Authors:** Xiawen Li, Yu Zhou, Guanghui Zhang, Yingzhi Lu, Chenglin Zhou, Hongbiao Wang

**Affiliations:** ^1^Department of Physical Education, Shanghai University of Medicine & Health Sciences, Shanghai, China; ^2^School of Psychology, Shanghai University of Sport, Shanghai, China; ^3^Center for Mind & Brain, University of California, Davis, Davis, CA, United States

**Keywords:** methamphetamine addiction, emotion, P1, EPN, LPP

## Abstract

**Background:**

Methamphetamine addicts can experience severe emotional processing disorders, with abnormal responses to emotional and drug-related stimuli. These aberrant behaviors are one of the key factors leading to relapse. Nevertheless, the characteristics of addicts’ responses to drug-related stimuli and their responses to emotional stimuli remain controversial.

**Methods:**

52 methamphetamine addicts from China passively viewed three different categories of images: Drug-related; positive emotional; and negative emotional. In the first task, participants completed a 9-point Self-Assessment Manikin (SAM) scale, rating the valence of each image. In the second, they performed a cued-action task while electroencephalography (EEG) data were recorded.

**Result:**

Drug-related images were rated negatively, with an average rating of 3.57. However, reaction times to drug-related stimuli were significantly faster than for negative stimuli (*p* = 0.030), and were indistinguishable from positive stimuli (*p* > 0.99). Similarly, EPN amplitudes evoked by drug-related images were significantly larger than those evoked by negative stimuli (*p* < 0.001), but no different than positive stimuli (*p* > 0.99). LPP amplitudes evoked by drug-related stimuli were significantly smaller than those evoked by negative (*p* < 0.001) and positive stimuli (*p* = 0.004).

**Conclusion:**

Despite negative self-assessments of drug-related imagery, MA-addicts reaction times were no slower than positive reactions. Similarly, drug-related and positive imagery EPN amplitudes were indistinguishable. Together, these results suggest increased attentional resources were allocated to the processing of drug-related stimuli and the pathways responsible partially overlap with the those recruited in processing positive emotional imagery in addicts. Moreover, in the late stage of visual processing, MA-addicts showed reduced brain activity in response to drug-related stimuli, suggesting reverse inhibition in response preparation and emotional appraisal. These findings may provide a reference for clinicians treating drug-taking behavior and for the development of new models of rehabilitation therapy.

## Introduction

Methamphetamine (MA) is a synthetic drug whose use has become increasingly prevalent in many countries, causing great harm to society, families, and individuals. Evidence shows that persons with MA dependency have higher rates of suicide and comorbidity, and are more likely to commit crimes (Marshall and Werb, 2010; Argento et al., 2017; Liu et al., 2017). Addicts often exhibit both impulsive and compulsive behaviors. In particular, they engage in compulsive drug taking despite the desire to stop taking the drug (Degoulet et al., 2021). Even after long periods of withdrawal treatment, once re-exposed to the drug environment, addicts are prone to relapse (Bechard and Knackstedt, 2019). Tackling these aberrant behaviors is key to the successful treatment of drug addiction.

Addicts also show severe emotional processing disorders, often experiencing abnormal emotional responses (Rabin et al., 2021; Warlow and Berridge, 2021). For example, long-term use of MA may lead to dysfunction of neural circuitry in the prefrontal and limbic systems (Feng et al., 2018). As a consequence, addicts show reduced arousal in response to non–drug-related positive emotional stimuli (May et al., 2020) and, concurrently, must increase the dose of the drug to maintain the same degree of pleasure (Volkow et al., 2011, 2016). Addicts may also have greater sensitivity to non–drug-related negative emotional stimuli, thus displaying higher incidences of depression and moodiness (Moustafa et al., 2020), which may lead to relapse. Therefore, if we wish to prevent relapse, understanding behavioral and neurological responses to emotional stimuli is paramount.

From an evolutionary perspective, human behavior is motivated by appetitive and defensive systems (Bradley et al., 2001; Lang and Bradley, 2010). Put simply, people approach positive stimuli and avoid negative stimuli. Compulsive drug taking can be considered an approach behavior, thus one might expect the addict’s response to drug-related stimuli to be consistent with their response to positive stimuli. However, long-term use of MA can cause addicts to experience strong negative emotions such as anxiety, depression, and anger (Alamdarloo et al., 2019; May et al., 2020). In addition, addicts learn about the dangers of drugs through drug-related laws and regulations, and social evaluations (Liu et al., 2018). Over time, these factors may cause addicts to react adversely to drug-related stimuli. Indeed, drug addicts tend to rate drug-related stimuli negatively (Wang et al., 2015). Given this discrepancy, further research is needed to elucidate the mechanisms underlying addicts’ responses to drug-related stimuli.

In recent years there has been a proliferation of research examining the neural processing of drug-related stimuli. For example, studies using functional magnetic resonance imaging (Costumero et al., 2018) and electroencephalography (EEG) (Motlagh et al., 2017; Bu et al., 2019) have demonstrated that compared with healthy controls, drug-dependent individuals show enhanced brain activity while looking at images associated with drugs. Moreover, adjacent research has found that brain activity elicited by drug-related stimuli is enhanced compared with that induced by general emotional stimuli (Marianne and Franken, 2011; Yang et al., 2015). This has not been without controversy, however, and other research has observed significant differences in brain activity only between drug-related stimuli and neutral stimuli, not between drug-related stimuli and emotional stimuli (Francesco et al., 2015).

In the present study, we investigated behavioral and neural responses to drug-related stimuli using a Self-Assessment Manikin (SAM) scale, and a cued-action task in conjunction with EEG. More specifically, we assessed the difference between responses to drug-related stimuli and non–drug-related positive or negative emotional stimuli, in MA-addicts who were currently MA-abstinent. Participants completed a SAM scale and a cued action task (the latter while wearing EEG electrodes) after passively viewing images that were either drug-related or non–drug-related emotional images. Previous studies have shown negative emotion slows reaction times, whereas positive emotion leads to faster reaction times (Beatty et al., 2016; Li et al., 2019). We analyzed EEG event-related potentials (ERPs), including P1, early posterior negativity (EPN), and the late positive potential (LPP). These three components are closely related to emotional stimulus processing, with ERP amplitudes previously associated with emotional stimuli (Aftanas et al., 1998; Flaisch et al., 2010; Lu et al., 2021). We hypothesized that both MA-addicts’ behavioral and neural responses to drug-related stimuli would differ from those associated with non–drug-related emotional stimuli.

## Materials and Methods

### Participants

In total, 52 MA-addicts (with 3 months of abstinence) were enrolled through the Drug Rehabilitation Bureau of Zhejiang Province. All participants were native Chinese men aged between 18 and 45 years, met the *Diagnostic and Statistical Manual of Mental Disorders* (Fifth Edition) criteria for a current MA use disorder, had no self-reported history of mental illness or chronic physical illness, were right-handed, and had normal or corrected-to-normal vision. Table 1 summarizes the demographic characteristics of the participants.

**TABLE 1:** Demographic and drug-taking characteristics of the study sample.

Variable	Mean (*SD*)
Age (years)	34.7 (6.7)
Educational level (years)	8.7 (1.7)
Methamphetamine dependency (years)	6.1 (3.5)
Frequency of use (day/week)	2.6 (2.2)
Amount of use (gram/dose)	0.4 (0.4)

*Drug-taking habits reflect those prior to rehabilitation and abstinence.*

The present study was approved by the ethics committee of Shanghai University of Sport (No. 102772019RT041) and conducted in accordance with this approval. Written, informed consent was obtained from all participants prior to their participation and confidentiality was upheld at all times.

### Materials

The present study used 195 images, of which 130 images were selected from the Chinese Affective Picture System (65 images were associated with positive emotions, and 65 with negative emotions) (Lu et al., 2005) and 65 MA-related images were selected from available online sources. All images were aligned in size and luminance. The formal experiment utilized 180 images (60 positive, 60 negative, and 60 MA-related), with the remaining 15 used to familiarize participants with instructions.

The images were assessed for valence and arousal on a 9-point Self-Assessment Manikin (SAM) scale by 30 MA-addicts who were not participants in the formal experiment to assess the suitability of our SAM measure (Table 2).

**TABLE 2:** The SAM ratings of emotional pictures.

Emotion type	Valence *M* (SD)	Arousal *M* (SD)
Negative	2.78 (1.10)	6.37 (1.35)
Positive	6.66 (1.15)	5.30 (0.96)
MA-related	3.30 (1.63)	4.30 (1.98)

Two one-way repeated measures ANOVAs were conducted to compare valence and arousal, respectively, between the three levels of emotional stimuli. We found there was an effect of emotion type on valence [*F*_(2, 28)_ = 62.836, *p* < 0.001]. More specifically, *Post hoc* pairwise *t*-tests (Bonferroni corrected) revealed positive (*M* = 6.66, *SD* = 1.15) emotional stimuli had greater valence than negative (*M* = 2.78, *SD* = 1.10) stimuli (*p* < 0.001) and MA-related (*M* = 3.30, *SD* = 1.63) stimuli (*p* < 0.001). In addition, there was no significant difference between the valence of the MA-related stimuli and the valence of the negative stimuli (*p* = 0.176). We also found an effect of emotion type on arousal [*F*_(2, 28)_ = 12.899, *p* < 0.001]. *Post hoc* pairwise *t*-tests (Bonferroni corrected) revealed negative (*M* = 6.37, *SD* = 1.35) stimuli had higher arousal ratings than positive (*M* = 5.30, *SD* = 0.96) stimuli (*p* < 0.05), which, in turn, had significantly higher arousal ratings than MA-related (*M* = 4.30, *SD* = 1.98) imagery (*p* < 0.05). The difference between positive and MA-related stimuli was not significant (*p* = 0.056).

### Design and Procedure

The experiment consisted of two tasks, both of which were a repeated-measures design with stimulus type (positive, negative, and MA-related images) as the factor. Each condition consisted of 60 images.

**FIGURE 1:**
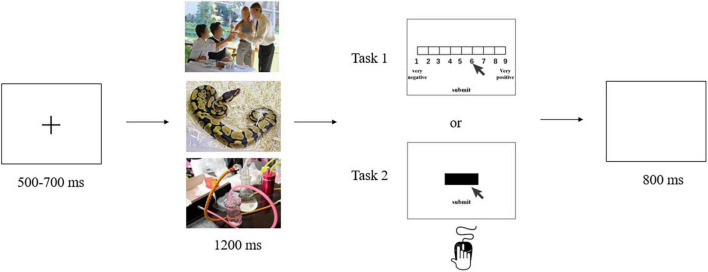
The 9-point SAM rating task (task 1) and the cued-action task (task 2).

For task 1, each trial began with a fixation cross presented on the computer screen for between 500 and 700 ms, drawn randomly from a uniform distribution. This screen was followed by a 1,200 ms presentation of one of the three image types. Participants were instructed to look carefully at the image. After the image disappeared, participants were asked to rate the valence of the image on a 9-point SAM scale using the keyboard (1 = very unpleasant; 9 = very pleasant). A blank screen was then presented for 800 ms before concluding the trial (Figure 1). The order of stimulus presentation was random.

Task 2 combined electroencephalography (EEG) with a cued-action task. Participants were seated approximately 60 cm from a computer in a dimly lit, quiet room. After participants had been introduced to the experiment, EEG electrodes were attached to them. During the experiment, a fixation cross was presented on the computer screen for between 500 and 700 ms, drawn at random from a uniform distribution. This screen was followed by a 1,200 ms presentation of one of the three image types. After the image disappeared, a black bar appeared on the screen to cue participants that an action was required. Participants were instructed to click on the black bar as soon as possible using their mouse, and then click a button labeled “submit.” A blank screen was then presented for 800 ms (Figure 1). Once again, the stimulus presentation order was random. The task procedure was compiled and run using E-Prime 2.0 software.

### Behavioral Data Acquisition and Analyses

Behavioral data consisted of rating scores from the 9-point SAM scale and reaction times from the cued-action task. Reaction times were computed as the time elapsed between when the black bar appeared on the screen, and when the participant clicked on it. Rating scores and reaction times were analyzed by one-way repeated-measures analyses of variance (ANOVAs) with image type (positive, negative, and MA-related) as main effects. *Post hoc* pairwise *t*-tests (Bonferroni corrected) were then run on any significant main effect. Statistical analyses were performed in SPSS, version 22.0 (IBM Inc.).

### Electroencephalography Data Acquisition and Analyses

EEG data from task 2 was collected with 64 Ag-AgCl electrodes arranged according to the international 10–20 system, with a sampling frequency of 1,000 Hz (Brain Products GmbH 64, Germany). The EEG was recorded referentially against the FCz electrode, and AFz served as the ground electrode. The vertical electrooculogram was recorded infraorbitally at the left eye, and the horizontal electrooculogram was recorded lateral to the orbit of the right eye. All electrooculogram and EEG electrode impedances were maintained at less than 5 kΩ.

Time-domain analyses were used to analyze the EEG data. We processed EEG data using a Brain Vision Analyzer2 (Germany). FCz was re-referenced to the average of the TP9 and TP10 electrodes. Ocular artifacts were then removed through ocular correction. Next, we removed line noise with a 50-Hz notch filter. Then, the data were filtered with a 30-Hz low-pass cutoff and a 0.5-Hz high-pass cutoff. The data were then segmented from 200 ms prior to the onset of the image to 1,000 ms after the image onset. All epochs were baseline-corrected with respect to the mean voltage over the −200 to 0 ms period preceding image onset and then averaged by experimental condition. Trials with amplitudes exceeding ± 80 μV were considered artifacts and thus excluded. P1, EPN, and LPP were selected as target ERP components. We analyzed the averaged P1 amplitude at the occipital-temporal electrodes PO7, PO8, O1, and O2 within the time window of 130–170 ms, the averaged EPN at electrodes PO7 and PO8 within the time window of 180–240 ms, and the averaged LPP at the Cz and CPz electrodes within the time window of 500–1,000 ms. One-way repeated-measures ANOVAs were used to analyze the averaged amplitude of P1, EPN, and LPP with image type (positive, negative, and MA-related) as the experimental condition.

## Results

### Task 1: Valence Scores

**FIGURE 2:**
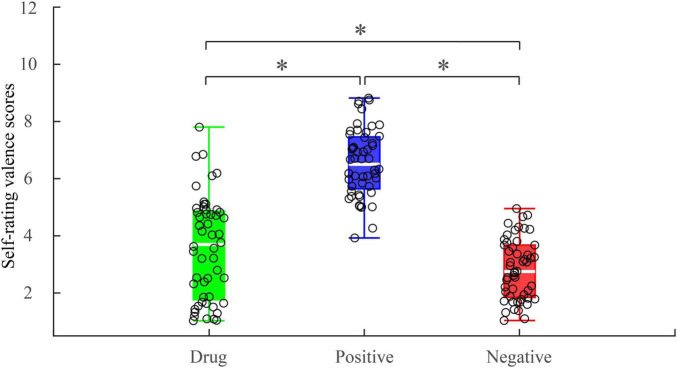
Self-rating valence scores under different experimental conditions. *p < 0.05.

A one-way repeated measures ANOVA showed a main effect of image type [*F*_(2, 50)_ = 120.436; *p* < 0.001; η*_*p*_*^2^ = 0.828] on reported valence. *Post hoc* pairwise comparisons indicated valence scores associated with MA-related images (*M* = 3.57, *SD* = 1.78) were significantly higher than those for the negative images [*M* = 2.82, *SD* = 1.05] (*p* = 0.004) and significantly lower than those for the positive images (*M* = 6.54, *SD* = 1.18, *p* < 0.001) (Figure 2). Importantly, while MA-related images were rated higher than negative images, the mean value was considerably lower than the median, suggesting MA-related images were emotionally negative stimuli for MA-addicts.

### Task 2: Reaction Times

**FIGURE 3:**
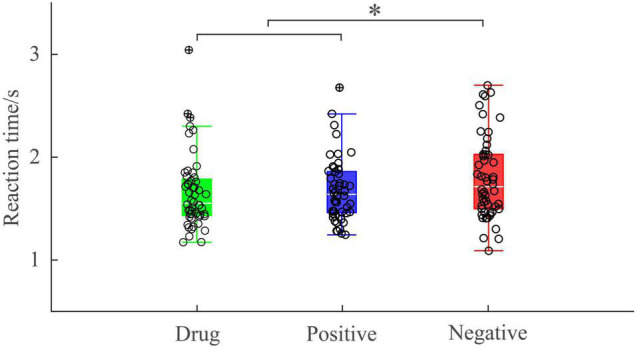
Reaction time under different experimental conditions. *p < 0.05.

A one-way, repeated-measures ANOVA showed there was a significant main effect of image type [*F*_(2, 50)_ = 4.635; *p* = 0.014; η*_*p*_*^2^ = 0.156] on reaction time. *Post hoc* pairwise comparisons indicated reaction times were significantly longer following negative images (*M* = 1801.57, *SD* = 401.61) than positive images (*M* = 1657.24, *SD* = 359.04, *p* = 0.036) and MA-related images (*M* = 1676.56, *SD* = 304.66, *p* = 0.030). There was no significant difference between the reaction time after positive images and MA-related images (*p* > 0.99) (Figure 3). This suggests MA-addicts’ behavioral responses to drug images were consistent with their behavioral responses to positive emotional images.

### Task 2: Electroencephalography Results

#### P1 Results

**FIGURE 4:**
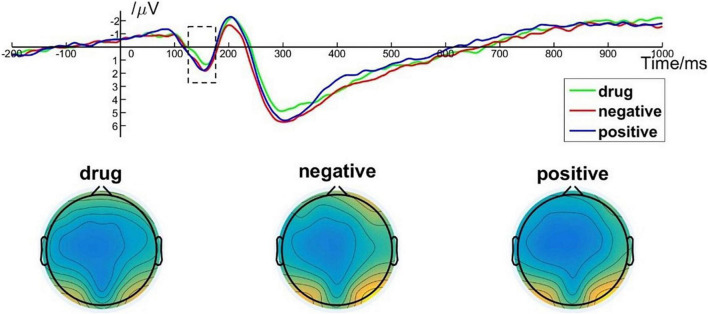
P1 component elicited under different conditions at electrode sites PO7, PO8, O1, and O2 and brain topography of P1.

One way, repeated measures ANOVA revealed a main effect of image type on the P1 amplitude [*F*_(2, 50)_ = 5.425; *p* = 0.007; η*_*p*_*^2^ = 0.178]. *Post hoc* pairwise comparisons indicated that the P1 amplitudes elicited by MA-related images (*M* = 0.90, *SD* = 2.22) were significantly smaller than those elicited by positive (*M* = 1.37, *SD* = 2.43, *p* = 0.049) or negative (*M* = 1.37, *SD* = 2.43, *p* = 0.017) images. There was no significant difference between P1 amplitudes elicited by positive images and negative images (*p* > 0.99). P1 brain topography illustrated that the P1 component was particularly robust in the occipital-temporal cortex (Figure 4). The P1 component has been shown to be closely related to early visual processing (Schupp et al., 2006; Zhang et al., 2014). Thus, this finding could suggest that the early visual processing of MA-related images differed in some way from the early processing of non–drug-related emotional stimuli.

#### Early Posterior Negativity Results

**FIGURE 5:**
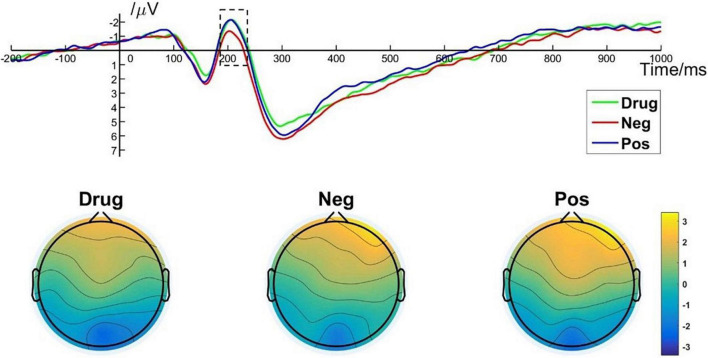
EPN component elicited under different conditions at electrode sites PO7, PO8, and brain topography of EPN.

One-way repeated measures ANOVA showed a significant main effect of image type [*F*_(2, 50)_ = 14.303; *p* < 0.001; η*_*p*_*^2^ = 0.364] on EPN amplitude. *Post hoc* pairwise comparisons indicated that the averaged EPN amplitude evoked by negative images (*M* = –0.49, *SD* = 3.17) was significantly lower than that evoked by MA-related images (*M* = –1.32, *SD* = 3.12, *p* < 0.001) and by positive images (*M* = –1.28, *SD* = 3.67, *p* < 0.001). There was no significant difference between the EPN amplitude evoked by MA-related images and positive images (*p* > 0.99). Brain topography illustrated the EPN was particularly robust in the occipital cortex (Figure 5). EPN amplitudes are closely related to the middle stage of specific image content processing (Zhang et al., 2014). Thus, these results indicate brain activity associated with the middle stage of drug-related image processing was indistinguishable from that associated with non–drug-related positive images.

#### Late Positive Potential Results

**FIGURE 6:**
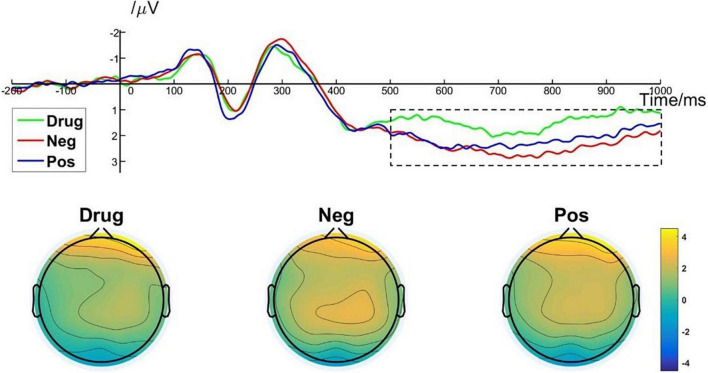
LPP component elicited under different conditions at electrode sites Cz, CPz, and brain topography of LPP.

A one-way repeated measures ANOVA showed a significant main effect of image type [*F*_(2, 50)_ = 10.478; *p* < 0.001; η*_*p*_*^2^ = 0.295] on LPP amplitude. More specifically, *post hoc* pairwise comparisons indicated that the average LPP amplitude evoked by MA-related images [*M* = 1.46, *SD* = 1.51] was significantly lower than that evoked by positive images (*M* = 2.15, *SD* = 1.45, *p* = 0.004) and by negative images (*M* = 2.40, *SD* = 1.50, *p* < 0.001). There was no significant difference in LPP amplitude between positive images and negative images (*p* = 0.513). Brain topography of the LPP illustrated it was particularly robust in the parietal cortex (Figure 6). The LPP amplitude is closely related to the evaluation of emotional stimuli and response preparation (Lang and Bradley, 2010; Yang et al., 2015). Thus, these results indicate that MA addicts may have reverse inhibition in their emotional appraisal and reaction preparation to drug-related stimuli (Moser et al., 2006).

## Discussion

The present study investigated behavioral and neural responses to drug-related and non–drug-related emotional stimuli in MA addicts. Previous research has investigated these responses in isolation. The present research represents the first time self-reports have been used in conjunction with reaction times and ERP characteristics to explore this question. We found behavioral and brain response patterns to drug-related stimuli differed from those for processing non–drug-related emotional stimuli. More specifically, participants reported the valence of drug-related images was significantly lower (higher) than positive (negative) stimuli. Moreover, drug-related images corresponded to reaction times that were indistinguishable from the response to positive images, and significantly quicker than the response to negative images. This relationship was mirrored in EPN response amplitudes, with responses to MA-related imagery evoking larger amplitudes than negative imagery, that were indistinguishable from responses to positive stimuli. We also found evidence for reverse inhibition in action preparation and emotional appraisal to drug stimuli in MA-abstinent addicts.

Stimulants such as methamphetamine are characterized by their ability to elicit rapid euphoria. Studies have shown that such drugs are perceived as more arousing and pleasant among people who are drug-dependent than people who are not (Jayanthi et al., 2021). Nevertheless, there is also evidence drug addicts tend to rate drug-related stimuli as negative (Yang et al., 2015). In the present study, although the self-assessment scores on the emotional valence of the MA-related stimuli were significantly higher than those on the negative stimuli, participants gave a negative evaluation to the drug-related stimuli. This negative assessment may be due negative consequences of the drug. For example, negative emotional experiences after the direct effects of the drug has worn off (May et al., 2020), or from punishment by incarceration. Laws and regulations unambiguously define drug taking and trafficking as illegal acts. In turn, individuals are all too aware of the dangers of drug use, perhaps eliciting negative evaluations of them.

Despite negative self-assessments of drug-related stimuli, our behavioral data demonstrates that MA-dependent participants’ reaction times after viewing non–drug related negative emotional images were significantly slower than those after viewing MA-related images or non–drug related positive images. By contrast, there was no significant difference between reaction times after viewing MA-related images and non–drug related positive images. This aligns with previous studies which found that performance after viewing negative non–drug related stimuli were significantly slower (Li et al., 2019), whereas performance under positive emotions were significantly improved (Prével et al., 2021). Pereira et al. (2006) used four consecutive behavioral experiments to show that when positive, neutral, or negative emotional images were presented randomly or when similar types of images were presented in groups, the reactions of participants to positive images were significantly faster than their reactions to negative images (Pereira et al., 2006). Li et al. (2019) combined EEG with a cued-action task and found that negative stimuli significantly slowed reaction time, largely due to negative emotional processing consuming more resources than non-emotional processing, with this interference effect mainly occurring in the late action preparation phase (Li et al., 2019).

The present study examined ERP components (P1, EPN, and LPP) that have been found to be sensitive to emotional processing (Lang and Bradley, 2010; Luis, 2014). The P1 component is evoked in the occipital lobe and is associated with attentional resources for early visual processing. Previous studies have observed an enhanced amplitude of the P1 component elicited by drug- or alcohol-related stimuli compared with neutral stimuli in addicts (Kroczek et al., 2018), suggesting they have an attention bias to these stimuli. However, the present results showed that P1 amplitudes elicited by MA-related images were significantly smaller than those elicited by positive or negative images. The present result may be explained by mechanisms underlying the processing of novel stimuli. Based on the existence of a fast magnocellular circuit (Schönwald and Müller, 2014), P1 may reflect the automatic processing of visual stimuli and be related to a rapid extraction of the novel saliency of the information (Piccardi et al., 2020). For addicts, drug-related stimuli may be considered routine and ordinary, whereas emotionally charged images such as fires, car crashes, and beautiful scenery could be construed as more novel. In addition, previous studies have shown that people tend to have a negative stimulus bias. Evidence has shown that relative to neutral stimuli, emotional stimuli elicit larger P1 amplitudes (Rsg et al., 2019). Given we found no difference between P1 amplitudes for positive and negative images, perhaps the circuitry responsible for early emotional processing is impaired in MA addicts.

The occipital-temporal EPN exhibited a more negative deflection for MA-related images and images associated with positive stimuli, than with those elicited in response to negative images. In fact, we detected no significant difference between the EPN elicited by MA-related and positive imagery, indicating that the responses were indistinguishable. The EPN is typically considered an ERP component related to early selective attentional processing and specific content distinctions of emotional stimuli (Weinberg and Hajcak, 2010; Farkas et al., 2020). Schupp et al. (2006) identified the EPN as the first neural activity that reflects the emotional characteristics of a stimulus (Schupp et al., 2006). Some studies have shown that EPNs are larger for emotional stimuli than for neutral stimuli (Lang and Bradley, 2010). Consistent with our results, Francesco et al. (2015) demonstrated that relative to neutral stimuli, emotional stimuli enhanced the EPN over the occipital brain region and that the EPN was more pronounced for positive than for negative stimuli. Moreover, similar to positive stimuli, cigarette-related stimuli elicited a larger EPN than did negative stimuli (Francesco et al., 2015). However, there are also studies showing that heroin-related images elicit a larger EPN than both negative and positive images (Yang et al., 2015). In the present study, the similarity between MA-related and positive imagery elicited EPN amplitudes may suggest that the brain processing pathways for drug-related stimuli partially overlap with the processing pathways for positive emotional stimuli in addicts.

Another signature of the ERP sensitive to emotional processing examined in the present study was the centroparietal LPP, a component thought both to reflect the in-depth evaluation and motivational relevance of emotional stimuli, and be related to response preparation (Lang and Bradley, 2010; Yang et al., 2015; Kaunhoven and Dorjee, 2021). Our results showed that LPPs elicited by MA-related stimuli were significantly smaller than those elicited by negative or positive stimuli, whereas there was no significant difference between positive and negative stimuli. Yang et al. (2015) used an emotional Stroop task to explore differences in emotional processing between addicts and non-addicts. Their LPP results are consist with those in the present study. We speculate that the smaller LPP associated with MA-related stimuli may be closely related to the current situation of the participants in the present study. That is, they may inhibit their motivation for approaching drug-related stimuli because they are in compulsory isolation and are receiving anti-drug education, which may be understood as response inhibition. Moser et al. (2006) found that when participants were asked to actively suppress their responses to negative images of high arousal, there was a decrease in the LPP amplitude (Moser et al., 2006). However, inconsistent with our findings, previous studies have found that the amplitude of the LPP induced by drug-related stimuli is greater than that for general emotional stimuli (Dunning et al., 2011), suggesting that addicts may devote more cognitive resources to drug-related stimuli and that their motivation toward drugs is stronger. In addition, previous studies assessing people without drug addiction have found that the LPP evoked by negative stimuli was significantly larger than that evoked by positive stimuli. From an evolutionary perspective, people would instinctively react stronger to negative stimuli that may threaten them (Quiñones-Camacho et al., 2018). On the contrary, given we found no difference in LPPs evoked by negative and positive stimuli, perhaps chronic methamphetamine abuse damages the neural circuits associated with emotional processing in addicts and reduces their sensitivity to non–drug-related emotional stimuli.

## Conclusion

In conclusion, at the behavioral level, despite negative self-assessments of drug-related imagery, MA-addicts reacted as quickly to drug-related imagery as they did to positive imagery. At the neural level, we found evidence for increased attentional resource allocated to the middle stage of drug imagery processing. In the late stage of drug imagery processing, MA-addicts showed reduced brain activity associated with drug-related stimuli, suggesting a reverse inhibition in action preparation and emotional appraisal to drugs. These findings may provide a reference for clinicians treating drug-taking behavior and for the development of new models of rehabilitation therapy.

## Data Availability Statement

The raw data supporting the conclusions of this article will be made available by the authors, without undue reservation.

## Ethics Statement

The studies involving human participants were reviewed and approved by the Ethics Committee of Shanghai University of Sport (No. 102772019RT041) and conducted in accordance with this approval. The patients/participants provided their written informed consent to participate in this study.

## Author Contributions

XL, CZ, and HW designed and conducted the experiments. XL and YZ contributed to the acquisition of data. GZ, YL, and XL analyzed the data. XL and HW wrote the manuscript. XL and CZ provided critical revision of the manuscript for important intellectual content. All authors critically reviewed content and approved final version for publication.

## Conflict of Interest

The authors declare that the research was conducted in the absence of any commercial or financial relationships that could be construed as a potential conflict of interest.

## Publisher’s Note

All claims expressed in this article are solely those of the authors and do not necessarily represent those of their affiliated organizations, or those of the publisher, the editors and the reviewers. Any product that may be evaluated in this article, or claim that may be made by its manufacturer, is not guaranteed or endorsed by the publisher.
