# Corrigendum: Behavioral and brain reactivity associated with drug-related and non-drug-related emotional stimuli in methamphetamine addicts

**DOI:** 10.3389/fnhum.2022.1032007

**Published:** 2022-09-14

**Authors:** Xiawen Li, Yu Zhou, Guanghui Zhang, Yingzhi Lu, Chenglin Zhou, Hongbiao Wang

**Affiliations:** ^1^Department of Physical Education, Shanghai University of Medicine & Health Sciences, Shanghai, China; ^2^School of Psychology, Shanghai University of Sport, Shanghai, China; ^3^Center for Mind & Brain, University of California, Davis, Davis, CA, United States

**Keywords:** methamphetamine addiction, emotion, P1, EPN, LPP

In the published article, there was an error in affiliations 1 and 2. Instead of “1 Department of Physical Education, University of Medicine and Health Sciences, Shanghai, China, 2 School of Psychology, University of Sport, Shanghai, China,” it should be “1 Department of Physical Education, Shanghai University of Medicine & Health Sciences, Shanghai, China, 2 School of Psychology, Shanghai University of Sport, Shanghai, China.”

The authors apologize for this error and state that this does not change the scientific conclusions of the article in any way. The original article has been updated.

## Publisher's note

All claims expressed in this article are solely those of the authors and do not necessarily represent those of their affiliated organizations, or those of the publisher, the editors and the reviewers. Any product that may be evaluated in this article, or claim that may be made by its manufacturer, is not guaranteed or endorsed by the publisher.

